# Development and initial validation of a dog quality of life instrument

**DOI:** 10.1038/s41598-022-16315-y

**Published:** 2022-07-28

**Authors:** Amandine Schmutz, Nathaniel Spofford, Walter Burghardt, Geert De Meyer

**Affiliations:** 1 Waltham Petcare Science Institute, Melton Mowbray, UK; 2 Banfield Pet Hospital, Vancouver, WA USA; 3 Unaffiliated, San Antonio, TX USA

**Keywords:** Animal behaviour, Data acquisition, Quality of life

## Abstract

The increasing attention for the dog-owner relationship combined with advances in nutrition and veterinary care have made wellbeing a focal point for dog owners, veterinarians, and dog product and service providers. While canine wellbeing can be quantified by survey-based quality of life instruments like those used in human healthcare, there are currently few instruments available that can do this reliably and at scale. Here we report the development and initial validation of a canine quality of life instrument specifically designed to quantify wellbeing in the general dog population. The instrument is based on a simple 32-question survey and includes 5 daytime domains (energetic, mobile, relaxed, happy, sociable) and 3 mealtime domains (relaxed, interested and satisfied). It captures specific health-related aspects as well as more general wellbeing aspects and, in an initial sample of 2813 dogs, already provides useful insights on canine wellbeing. We believe that data collection at scale with this instrument will help bring optimal wellbeing to the dogs we care for.

## Introduction

Dogs have played a key role in society for centuries, over which the nature of dog ownership has continually evolved. The past decade has seen a particularly dramatic shift with owners increasingly seeing their dog as a family member^1^ and seeking out products and services like those they would use for themselves^2^. In parallel, advancements in veterinary care have reduced the prevalence of communicable disease and made chronic conditions such as periodontal disease and obesity the most common health conditions^3,4^. Such conditions are not easily resolved and require management through ongoing care and support. Because of these evolutions, wellbeing has become of major interest to dog owners, veterinarians, and a wide range of dog product and service providers^5–8^.

As opposed to animal welfare that focuses on the five freedoms as essential life requirements, we use wellbeing to denominate the varying levels of quality that make a life worth living^9^ across multiple domains including diet, environment, company, human interactions, and health^10^. Quality of life (QoL) can be objectively quantified by survey-based instruments that are developed and validated following standard psychometrics methodology and are widely used in human healthcare^11^. There are also canine QoL instruments for which owners fill out the survey based on their observations^12–16^. These instruments are typically developed in small studies (n = 100 to 200) of specifically selected dogs, which leaves as an open question to what extent these instruments capture all wellbeing aspects in the general population. Additionally, many canine QoL instruments focus on specific health conditions or certain aspects of QoL (e.g. health related QoL), which limits their use to niche research applications or to clinical applications, respectively. As a result, there is an opportunity to develop a QoL instrument that reliably quantifies the full range of wellbeing in the general canine population.

The primary objective of this study is to develop a comprehensive canine QoL instrument that can serve a wide range of applications and is amenable to large scale data collection. In addition, we aim to demonstrate instrument validity and conduct an initial characterization of wellbeing in a large sample of dogs.

## Materials and methods

### Survey design, initial questionnaire development, and data collection

Surveys were structured as a sequence of identically phrased questions to the dog owner with each question or item addressing a specific dog behavior. We used two lead questions: “Please tell us how well each of these words describe your dog as he/she is today” and “please tell us how well each of these words describe your dog at mealtime” to collect daytime and mealtime information respectively. Items were scored on a 1 to 7 Likert scale with 1 labeled as “does not describe at all” and 7 labeled as “very much describes”. Technical implementation ensured that completed surveys had no missing data.

The initial item set to probe QoL across multiple domains was developed by a five-person team consisting of pet owners, veterinarians, veterinary nutritionists, and veterinary behaviorists. A literature review identified 9 commonly used QoL domains addressing physical (energy, mobility, pain, appetite, hydration, hygiene), emotional (happiness, anxiety) and social aspects (social interaction). Tentative mappings for domains from 4 key publications^12–15^ onto this set are shown in Supplementary Table S1 as an example. The team decided to exclude two of these domains for the initial item set development: pain because its effect is likely reflected in the other domains^10^ and hygiene because it did not pass validation^15^. For the remaining 7 domains of interest the team then developed an item set consisting of words that based on their experience and on inclusion in existing canine QoL instruments^12^ provide direct or indirect information on these domains. Because the team felt that general appetite questions might lack resolution a specific mealtime section was added to the survey as a potentially better practical way to obtain this information. This resulted in a 94-item questionnaire with 52 daytime items and 42 mealtime items (Supplementary Table S2).

The initial 94-item version of the survey was piloted on MARS employees from all United Kingdom and United States sites via internal social media groups. Responses were collected over two weeks (employee study). A second 98-item version (see Supplementary Table S2 for included items) was sent out by e-mail to 3929 participants of the Pet Insight Project^17^ and responses were collected over a week (citizen science study). The final data collection used a 36-item version (see Supplementary Table S3 for included items) and was executed by the Banfield Pet Hospital network of over 1000 general practice hospitals across the United States. A random sample of 49,000 dog owners received the survey by e-mail, and responses were collected over a week. Respondents were sent the same survey again 10 days after the initial contact to obtain data on survey repeatability (hospital client study). Given that the studies did not involve interventions on animals they were deemed exempt from ethical approval by the MARS ethical review board. Study objectives were shared prior to the survey and participants consented to these by completing the survey. The usage of electronic medical record data for scientific purposes (see below) is consented to by Banfield Pet Hospital clients.

### Deriving dog signalment information and chronic disease status from medical records

To support sample characterization and construct validity analyses, surveys from the citizen science and hospital client studies were linked to the dog’s electronic medical record. Basic signalment information including age, breed, and sex were extracted from the last available visit with age recalculated to the survey date. Breeds were recoded into size categories toy, small, medium, large, and giant based on the breed’s average adult body weight^18^. Age-based life stage coding into the categories youth, midlife, and senior used breakpoints at 7 and 11 years for toy and small dogs, and breakpoints at 6 and 10 years for medium, large and giant dogs. Body condition score (BCS) was extracted from the last visit when available and carried forward from previous visits when not. It was recoded into the categories underweight, normal, and overweight from the original 5- or 9-point scale. The underweight group was ignored for the analysis because it included only 9 dogs.

Chronic disease status was scored for 5 disease clusters: osteoarthritis, gastro-intestinal (GI) disease, cardiac disease, dental disease, and skin disease. A definition for each disease cluster was developed based on a set of structured diagnostic codes identified by a board-certified veterinary specialist as being associated with the condition. Osteoarthritis, GI disease, and cardiac disease were scored “present” when at least one associated diagnostic code was recorded during any visit in the medical record, and “absent” otherwise. For GI disease and skin disease, we further imposed the cluster diagnostic code to be recorded at least once in the 18 months prior to the survey to increase the probability that the disease was still present at the time of the survey. For skin disease we further added the requirement that a cluster diagnostic code was recorded during at least 3 different visits, again to enrich for chronic conditions.

### Data analysis

For instrument development and validation, a factor analysis was performed on all items in scope using the R package psych^19^. The initial number of factors was determined by parallel analysis^20^. Factors were sequentially reduced considering interpretability, by removing items with a too low (r < 0.30) or a too high (r > 0.8) within-domain Pearson correlation coefficient r, and by assessing item repeatability. For the latter the intraclass correlation coefficient^21^ was calculated on the subset of dogs for whom repeated surveys were available. For the final instrument development stage and for validation analyses the number of factors was fixed a priori. For some domains in the final instrument, factor loadings were reversed so that all domains have higher scores for increasing levels of the construct expressed by the domain name. In order to ease interpretation, domain scores were mapped back on the original 1–7 scale.

In the construct validity analyses, associations between domain scores and factors of interest were tested with a non-parametric Mann–Whitney U test and with a Kruskal–Wallis test in case of 2 or more than 2 factor levels, respectively. Factor effects were expressed as the difference between the factor level median and the median of the factor reference level. To enable effect size comparison between domains, factor effects were also scaled in approximate units of the domain’s population standard deviation. A robust estimate of the population standard deviation was obtained by dividing the domain interquartile range by 1.35, the number of standard deviation units the interquartile range covers in a normal distribution. Reported p values are not corrected for multiple testing but we only called statistical significance in case of a p value below 0.001. This corresponds to a Bonferroni correction for the 72 tests performed in this paper (0.05/72 ≈ 0.001) and guarantees the fraction of reported false positive results to be less than 7.2%. All analyses were performed in the statistical software R^22^ version 3.6.3 using standard functions and packages where not explicitly mentioned.

## Results

### Developing a comprehensive canine QoL instrument

The initial 94 item questionnaire was used to collect data in the employee and citizen science studies (Table 1) with 4 mealtime items added in the citizen science study based on insights from the employee study. Analysis of combined employee and citizen science studies by an iterative process of exploratory factor analysis, interpretation, and item pruning resulted in a 36-item core set with 21 daytime items and 15 mealtime items (Supplementary Table S3). More data on this core item set was then collected in the hospital client study (Table 1), leading to a final data set of 36 items scored for 2813 dogs.

**Table 1:** Characteristics and basic signalment information for the 3 studies performed. Summaries for continuous variables are given as mean (standard deviation).

Study name	Employee	Citizen science	Hospital client
Geography	United Kingdom and United States	United States	United States
Items tested	94	98	36
Sample size	–	3929	49,000
Respondents	310	868	1635
Response rate (%)	–	22.1	3.3
Repeat respondents	–	–	421
Dog breeds	–	105	120
**Dog size (%)**			
Toy	–	14.1	17.2
Small	–	25.9	27.4
Medium	–	21.9	21.7
Large	–	35.0	30.8
Giant	–	3.2	3.1
Dog age in years	–	7.3 (3.6)	6.5 (4.1)
**Dog life stage (%)**			
Youth	–	44.5	52.0
Midlife	–	23.0	28.4
Senior	–	32.6	19.6
Dog sex (% female–% male)	–	50.4–49.6	47.0–53.0
Respondents with medical record	–	854	1635
Number of visits in medical record	–	19.8 (14.6)	17.2 (14.1)
Time between survey and last visit (years)	–	0.27 (0.39)	0.21 (0.18)

**Figure 1:**
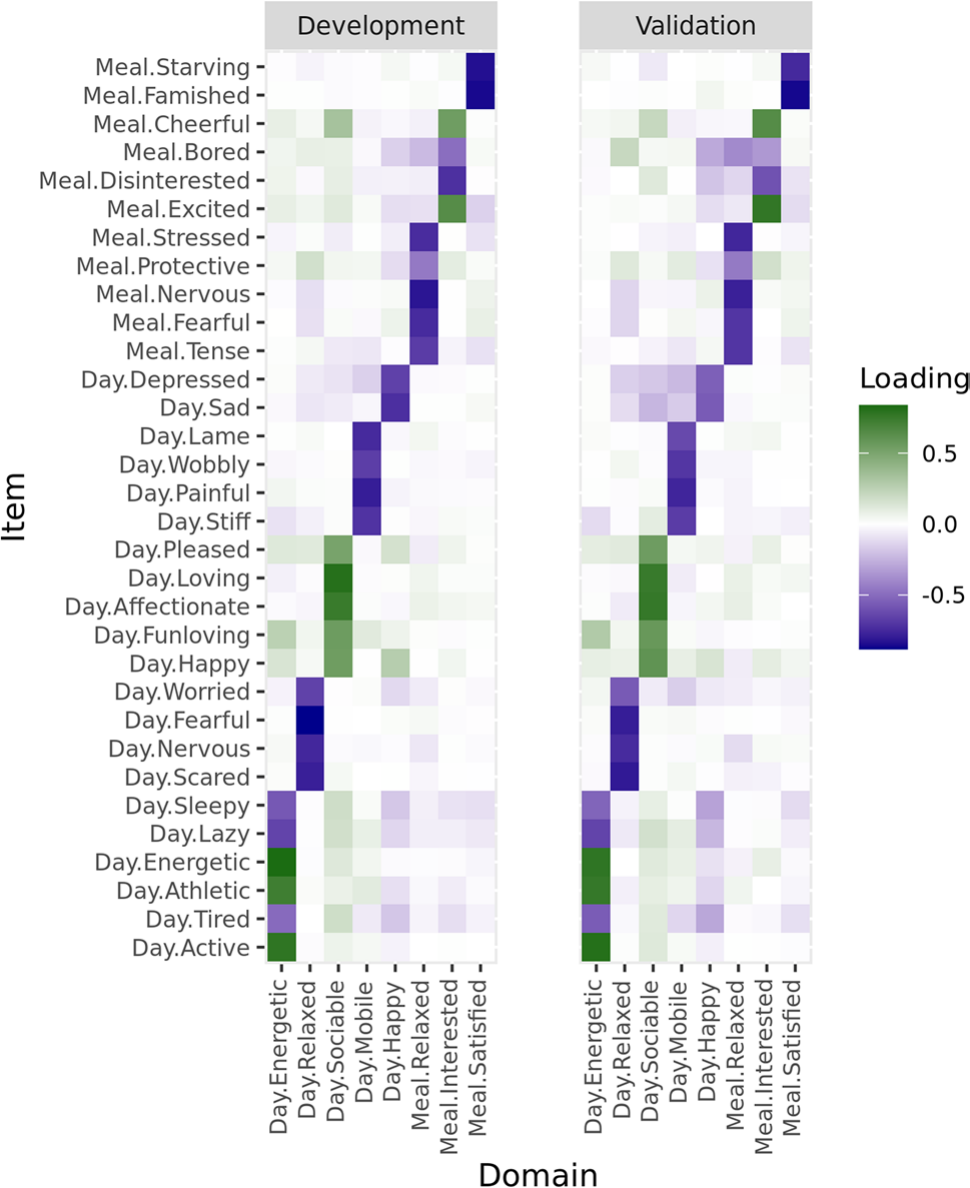
Instrument factor loadings and domain mapping obtained for instrument development (1996 surveys) and for an independent validation (817 surveys). Item and domain prefixes day and meal refer to daytime and mealtime, respectively.

The canine QoL instrument was ultimately developed on data from 1996 surveys and 36 items. This included all surveyed dogs from the employee and citizen science studies and 818 (50%) randomly selected surveyed dogs from the hospital client study. This allowed using a large sample with diversity in geography, breed, size, and life stage (Table 1) for instrument development, while keeping 817 surveyed dogs from the hospital client study for independent validation. Analysis of these data resulted in an 8-domain instrument based on 32 items (Fig. 1). Domains are primarily based on 2 to 6 items with an absolute loading above 0.50 that have moderate (0.30 < r < 0.80) within-domain correlations. Based on 213 out of the 818 surveyed dogs from the hospital client study for whom replicates are available, item repeatability in terms of the intra-class correlation coefficient ranged from 0.36 to 0.71, with 21 out of 32 items having an intra-class correlation coefficient above 0.50. The instrument has five daytime domains that are primarily based on daytime items. Two daytime domains, energetic and mobile, are linked to physical activity with energetic reflecting the level of activity and mobile the underlying mechanistic ability. Two other daytime domains describe the dog’s perceived emotional state: the relaxed domain manifests as a general calmness and absence of fear and worry while the happy domain reflects as the absence of a sad and depressed demeanor. The last daytime domain, sociable, is primarily driven by affectionate and loving behavior towards owners and other pets. The three mealtime domains reflect calm behavior around the meal as evidenced by the absence of stress (relaxed), the interest and excitement for the meal provided (interested), and the extent to which the dog is full or satisfied after the meal (satisfied).

### Supporting the instrument’s content validity and reliability

Two key elements of instrument validation, content validity and reliability, were assessed based on 817 surveyed dogs from the hospital client study that were not used for instrument development. While content validity, or the ability of a domain to represent the underlying concept, is intrinsically addressed by item selection and domain naming, we did verify domain structure consistency. An 8-domain factor analysis applied to the 32 selected items on the independent data (Fig. 1) revealed a domain structure with a nearly identical mapping of the main items (absolute factor loading above 0.5) onto the domains. There were only slight variations for the smaller loadings. Based on this consistency, that was also observed between the 3 studies when analyzed individually (Supplementary Fig. S1), and on the item selection logic we conclude that content validity is satisfactory. For the instrument’s reliability or the consistency of its scores, we used the 208 out of 817 dogs for which we had replicated results. Item repeatability assessed by the intra-class correlation coefficient ranged from 0.35 to 0.71 with most items, 22 out of 32, having a value above 0.50. The correlation coefficient between domain score repeats ranged from 0.54 to 0.81, which is satisfactory.

### Characterizing canine wellbeing and construct validity

**Figure 2:**
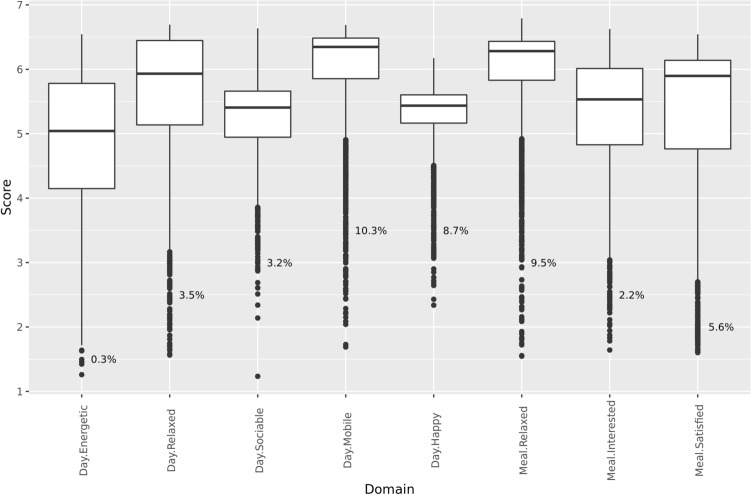
Domain score distribution for all surveyed dogs. Boxes delineate the 25th and 75th percentiles and show the median position in between. Whiskers indicate the range of all data excluding outliers and extreme values. The fraction of outliers and extreme values is represented explicitly as the dots representing these are overplotted.

Construct validity, or the extent to which a domain measures what it intends to measure, was assessed by the known groups approach^12,23^ that tests whether domain scores differ between groups with known wellbeing differences. To start, we explored the domain score distribution for all 2813 dogs surveyed (Fig. 2). With ranges spanning roughly from 1.5 to 6.5, domains generally have a good coverage of the scale. Domain variability differs markedly between domains with some, such as the energetic daytime domain, showing high variability and others, such as the happy daytime domain, less. Most domains have 5% to 10% outliers and extreme values in the low score range. This suggests distributions with most dogs having a relatively high and similar domain score and a minority of dogs displaying a high variability of extreme low scores.

For the 2489 dogs with a medical record, we studied how basic signalment information (Table 2) and chronic disease cluster status (Table 3) relate to domain scores. The two physical daytime domains energetic and mobile are affected by body condition score, life stage, and osteoarthritis with lower scores linked to overweight, older age, and disease. Osteoarthritis effects are larger for the mobile domain than for the energetic domain as evidenced by their scaled effects (Table 3) and are consistent across life stages (Supplementary Fig. S2). The energetic and mobile domains are also impacted by all other chronic disease clusters except GI disease. For the three emotional daytime domains, sociable and happy domain scores reduce with life stage and with presence of chronic disease except for GI disease, albeit that not all effects reach statistical significance for the happy domain. Presence of chronic dental disease is associated with lower scores in the relaxed daytime domain. For the mealtime domains, the interested and satisfied scores decrease with life stage. The interested mealtime domain is also reduced for dogs with chronic dental disease while satisfied mealtime domain scores are lower in dogs with osteoarthritis and likely lower for dogs with cardiac disease and chronic skin disease. Taken together, a multitude of expected associations support construct validity for all instrument domains except the relaxed mealtime domain.

**Table 2:** Effect of body condition score (BCS), sex, size and life stage on domain score.

	Day.Energetic			Day.Relaxed			Day.Sociable			Day.Mobile		
	Crude effect	Scaled effect	p value	Crude effect	Scaled effect	p value	Crude effect	Scaled effect	p value	Crude effect	Scaled effect	p value
**BCS**			< 0.001			0.947			0.761			< 0.001
Normal	0.00	0.00		0.00	0.00		0.00	0.00		0.00	0.00	
Overweight	− 0.54	− 0.44		0.00	− 0.01		− 0.01	− 0.02		− 0.13	− 0.40	
**Sex**			0.339			0.052			0.788			0.496
Female	0.00	0.00		0.00	0.00		0.00	0.00		0.00	0.00	
Male	0.12	0.10		0.09	0.17		− 0.04	− 0.08		0.00	0.00	
**Size**			0.005			0.269			0.002			0.150
Toy	0.00	0.00		0.00	0.00		0.00	0.00		0.00	0.00	
Small	0.03	0.03		0.17	0.31		0.06	0.13		0.04	0.11	
Medium	− 0.01	0.00		0.11	0.20		0.03	0.05		0.02	0.05	
Large	0.20	0.16		0.10	0.19		0.12	0.25		0.06	0.19	
Giant	0.02	0.01		0.19	0.36		0.11	0.24		− 0.07	− 0.20	
**Life stage**			< 0.001			0.072			< 0.001			< 0.001
Youth	0.00	0.00		0.00	0.00		0.00	0.00		0.00	0.00	
Midlife	− 0.67	− 0.55		− 0.10	− 0.19		− 0.10	− 0.22		− 0.16	− 0.49	
Senior	− 1.32	− 1.09		− 0.06	− 0.12		− 0.28	− 0.60		− 0.67	− 2.07	

	Day.Happy			Meal.Relaxed			Meal.Interested			Meal.Satisfied		
	Crude effect	Scaled effect	p value	Crude effect	Scaled effect	p value	Crude effect	Scaled effect	p value	Crude effect	Scaled effect	p value
**BCS**			0.584			0.240			0.707			0.022
Normal	0.00	0.00		0.00	0.00		0.00	0.00		0.00	0.00	
Overweight	0.01	0.01		− 0.04	− 0.04		0.01	0.03		− 0.17	− 0.20	
**Sex**			0.427			0.486			0.240			0.219
Female	0.00	0.00		0.00	0.00		0.00	0.00		0.00	0.00	
Male	− 0.01	− 0.01		− 0.02	− 0.02		0.04	0.10		− 0.01	− 0.01	
**Size**			0.094			0.220			0.109			0.095
Toy	0.00	0.00		0.00	0.00		0.00	0.00		0.00	0.00	
Small	0.00	0.00		0.05	0.06		0.00	− 0.01		0.03	0.03	
Medium	− 0.04	− 0.04		0.04	0.04		0.09	0.20		− 0.12	− 0.13	
Large	− 0.01	− 0.01		0.05	0.05		0.03	0.06		0.04	0.04	
Giant	− 0.12	− 0.12		0.06	0.07		0.03	0.07		− 0.20	− 0.23	
**Life stage**			< 0.001			0.308			< 0.001			< 0.001
Youth	0.00	0.00		0.00	0.00		0.00	0.00		0.00	0.00	
Midlife	− 0.06	− 0.06		0.00	0.00		− 0.06	− 0.13		− 0.22	− 0.26	
Senior	− 0.16	− 0.16		− 0.02	− 0.02		− 0.18	− 0.40		− 0.16	− 0.18	

Effects are expressed versus a reference category either on the 1 to 7 domain score scale (crude effect) or approximately scaled to the domain score population standard deviation (scaled effect).

**Table 3:** Effect of Osteoarthritis, gastro-intestinal (GI) disease, cardiac disease, dental disease, and skin disease on domain scores.

	Day.Energetic			Day.Relaxed			Day.Sociable			Day.Mobile		
	Crude effect	Scaled effect	p value	Crude effect	Scaled effect	p value	Crude effect	Scaled effect	p value	Crude effect	Scaled effect	p value
**Osteoarthritis**			< 0.001			0.163			< 0.001			< 0.001
Absent	0.00	0.00		0.00	0.00		0.00	0.00		0.00	0.00	
Present	− 0.93	− 0.77		− 0.06	− 0.12		− 0.19	− 0.41		− 0.86	− 2.66	
**GI disease**			0.760			0.099			0.530			0.653
Absent	0.00	0.00		0.00	0.00		0.00	0.00		0.00	0.00	
Present	− 0.03	− 0.02		− 0.13	− 0.24		− 0.01	− 0.02		0.03	0.09	
**Cardiac disease**			< 0.001			0.888			< 0.001			< 0.001
Absent	0.00	0.00		0.00	0.00		0.00	0.00		0.00	0.00	
Present	− 0.98	− 0.81		0.00	0.00		− 0.16	− 0.34		− 0.28	− 0.87	
**Dental disease**			< 0.001			< 0.001			< 0.001			< 0.001
Absent	0.00	0.00		0.00	0.00		0.00	0.00		0.00	0.00	
Present	− 0.75	− 0.62		− 0.23	− 0.44		− 0.19	− 0.41		− 0.21	− 0.64	
**Skin disease**			< 0.001			0.190			0.543			< 0.001
Absent	0.00	0.00		0.00	0.00		0.00	0.00		0.00	0.00	
Present	− 0.38	− 0.31		0.10	0.19		− 0.04	− 0.09		− 0.10	− 0.31	

	Day.Happy			Meal.Relaxed			Meal.Interested			Meal.Satisfied		
	Crude effect	Scaled effect	p value	Crude effect	Scaled effect	p value	Crude effect	Scaled effect	p value	Crude effect	Scaled effect	p value
**Osteoarthritis**			< 0.001			0.841			0.029			< 0.001
Absent	0.00	0.00		0.00	0.00		0.00	0.00		0.00	0.00	
Present	− 0.11	− 0.11		0.02	0.02		− 0.11	− 0.24		− 0.45	− 0.51	
**GI disease**			0.549			0.343			0.870			0.341
Absent	0.00	0.00		0.00	0.00		0.00	0.00		0.00	0.00	
Present	0.01	0.01		− 0.02	− 0.02		− 0.02	− 0.05		− 0.06	− 0.07	
**Cardiac disease**			0.001			0.757			0.263			0.002
Absent	0.00	0.00		0.00	0.00		0.00	0.00		0.00	0.00	
Present	− 0.10	− 0.10		0.02	0.02		− 0.10	− 0.23		− 0.38	− 0.44	
**Dental disease**			0.002			0.027			< 0.001			0.335
Absent	0.00	0.00		0.00	0.00		0.00	0.00		0.00	0.00	
Present	− 0.07	− 0.06		− 0.03	− 0.03		− 0.16	− 0.36		− 0.12	− 0.14	
**Skin disease**			0.003			0.737			0.545			0.002
Absent	0.00	0.00		0.00	0.00		0.00	0.00		0.00	0.00	
Present	− 0.08	− 0.07		0.01	0.01		0.08	0.18		− 0.24	− 0.27	

Effects are expressed versus a reference category either on the 1 to 7 domain score scale (crude effect) or approximately scaled to the domain score population standard deviation (scaled effect). The present category included 301 dogs for osteoarthritis, 86 for GI disease, 214 for cardiac disease, 608 for dental disease, and 335 for skin disease.

## Discussion

In this study we developed a comprehensive 8-domain canine QoL instrument with 5 daytime domains (energetic, mobile, relaxed, happy, sociable) and 3 mealtime domains (relaxed, interested and satisfied). As expected by the universal nature of wellbeing, similar daytime domains have been identified in other canine QoL instruments. For example, the daytime domains energetic, mobile, relaxed, and happy are also included in a canine health related QoL instrument^12^ albeit based on a different item set. Likewise, an instrument for healthy dogs^15^ includes happy, a physical domain that might comprise energetic and mobile, and a mental domain that might capture relaxed. This raises the question which instrument or set of domains is best suited to quantify canine wellbeing in the general dog population. We believe that sampling strategy and sample size of instrument development studies are key aspects to assess this. As opposed to a case–control sample^12^, the general population sample approach taken in this study has the advantages of unbiased detection of domains that address real variation in the population, of using domain loadings tuned to optimally capture that variation, and of enabling reliable domain scaling. However, given the heavily skewed distribution of many domain scores with only 5% to 10% of dogs driving the bulk of the variability in the low score range (Fig. 2), a large sample size is required for a general population sample to have sufficient power. Likely our sample of approximately 2000 dogs is appropriate as it includes 100 to 200 dogs to capture the low-end variation. Therefore, we are confident that this study not only identifies the relevant daytime domains but also extends the view on canine wellbeing by adding mealtime domains. Yet, as mealtime domains could potentially capture transient motivational states further studies will be required to establish the relative contribution of the mealtime domains to overall QoL. In all this makes our 8-domain canine QoL a valuable new instrument to quantify canine wellbeing.

For the instrument’s validation status, the item mapping logic and the domain structure robustness (Fig. 1, Supplementary Fig. S1) provide strong evidence to support content validity. Domain score repeatability estimates for surveys taken 2 weeks apart that range from r = 0.54 to r = 0.81 are good but not fully convincing as r = 0.70 is a typical benchmark^11^. It is possible that for some domains 2 weeks is too long for the domain score to be constant, which makes further short interval repeatability studies essential. The known groups approach based on basic signalment information (Table 2) and disease status for 5 chronic diseases with roughly 100 to 600 affected dogs depending on the disease (Table 3) provides strong evidence for construct validity. For all domains except the mealtime domain relaxed, scores confirm reported disease effects (e.g. reduced daytime energetic and mobile scores associated with osteoarthritis^24,25^), effects that can be inferred by simple logic (e.g. reduced scores for the mealtime interested domain for dogs with chronic dental disease), or common knowledge (e.g. reduced scores for most domains with life stage). As a note of caution, these population-level effects do not necessarily imply that the instrument will work at the level of individual dogs or will detect changes over time. Therefore, further studies with well-characterized dogs and prospective longitudinal data collection are still required. Yet all things considered, initial validity tests show clear promise and support the instrument being used in practical studies to further document its validity, for example by linking results to concurrent biological measurements, behavior analysis and clinical diagnosis.

Results obtained in this study already provide insights into canine wellbeing. All domains except the daytime energetic domain have a clearly skewed distribution with most of the population centered in the high score range and the remaining 5% to 10% displaying a wide range of low scores (Fig. 2). Therefore, efforts to improve general wellbeing might best focus on this minority group of dogs. A second observation is that, in addition to picking up specific chronic disease effects (discussed above), the daytime domains energetic, mobile and sociable as well as the mealtime domain satisfied are negatively impacted by most chronic diseases (Table 3). This suggests that these domains also capture general malaise or lack of wellbeing, possibly driven by underlying pain^10^. Given that the survey is filled in by the dog owner it is unclear whether this genuinely reflects the dog’s wellbeing or includes unconscious bias from the dog owner, for example based on the dog’s signalment and health status. However, given the importance of the owner’s perception of their pet’s wellbeing in deciding to seek or continue care this information nevertheless provides valuable insights. In any case these findings support the link between health and wellbeing and demonstrate that the instrument can quantify owner-perceived wellbeing effects that are critical in the management of chronic diseases.

Pending further positive validation results our plan is to deploy this instrument at a large scale taking advantage of the fact that its simple structure allows it to be completed in 3 to 5 min and makes it easy to integrate in a wide range of apps. At that scale it will be possible to develop granular reference ranges (e.g. life stage specific) allowing dog owners to benchmark and monitor their dog’s wellbeing and intervene when deemed necessary. We further contend that this instrument can improve veterinary care. A survey completed prior to examination can make the consultation more efficient and reveal issues that could otherwise have gone unnoticed, while routine post-visit data collection can provide information for outcome evaluation and support delivery of value-based care^26^. More generally the survey will help everyone involved in improving dog’s lives (pet owners, veterinarians, service providers, the pet food nutrition industry, etc.) ensure that their efforts bring optimal wellbeing to the dogs they care for.

## Supplementary Information

Supplementary Information.

## Data Availability

The datasets generated during and analysed during the current study are not publicly available due to restrictions imposed on medical record data usage. Datasets are available from the corresponding author on reasonable request provided that this request does not conflict with restrictions imposed on medical record data usage.
