# Supplementary material for: Development and initial validation of a dog quality of life instrument
Source: Sci Rep. 2022 Jul 28;12:12225. doi: 10.1038/s41598-022-16315-y (PMC9334304; doi:10.1038/s41598-022-16315-y)
Supplement: Supplementary file 1 — Supplementary Information. [file 41598_2022_16315_MOESM1_ESM.pdf]

Supplementary material

Manuscript title: Development and initial validation of a dog quality of life instrument.

Authors: Amandine Schmutz, Nathaniel Spofford, Walter Burghardt, and Geert De Meyer*

Supplementary Table S1. Mapping quality of life domains from different instruments onto a common scheme based on domain description and domain items. Some instrument domains map onto multiple common scheme domains and some domains of the common scheme span multiple instrument domains. References point to the main paper.

	Lynch et al. [14]	Iliopoulou et al. [13]	Lavan [15]	Reid et al. [12]
Energy	-	Play activity	Physical functioning	Energetic/ Enthusiastic
Mobility	Mobility	Mobility<br>Perceived activity	Physical functioning	Active/ Comfortable
Pain	Pain	Perceived pain	Physical functioning	-
Appetite	Appetite	Appetite	-	-
Hydration	Water intake	-	-	-
Hygiene	Hygiene	-	-	-
Happiness	Happiness<br>Mental status	Happiness<br>Joy	Happiness<br>Mental status	Happy/ Content
Anxiety	-	Anxiety	-	Calm/ Relaxed
Social interaction	Happiness	-	Happiness	-

Supplementary Table S2. Tentative mapping of the 52 daytime items and the 42 mealtime items included in the initial questionnaire onto the common domain scheme. Four items marked with an asterisk were added in the citizen science study.

Setting	Domain	Items
Daytime	Energy	Active, Athletic, Energetic, Enthusiastic, Fun-loving, Lazy, Lethargic, Playful, Reluctant to go out for a walk, Sleepy, Tired, Tired after a walk, Tired throughout the day, Unmotivated
	Mobility	Comfortable, Groaning, Lame, Limping, Painful, Reluctant to go up and downstairs, Sore, Stiff, Wobbly
	Appetite	Hungry, Nauseated, Scavenger
	Hydration	Have diarrhoea, Thirsty
	Happiness	Confused, Depressed, Happy, Listless, Pleased, Sad
	Anxiety	Agitated, Aloof, Calm, Fearful, Grumpy, Irritated, Nervous, Relaxed, Scared, Seems their normal self, Sensitive to touch, Worried
	Social interaction	Affectionate, Friendly, Independent, Loving, Reclusive, Responding to stimulation
Mealtime	Appetite	Aggressive, Ambivalent, Anticipating*, Anxious, Aversive, Avoidant, Begging*, Bored, Calm, Cheerful, Content, Concerned, Crazy, Curious, Disappointed, Disinterested, Distracted, Drooling, Excited, Excitable, Famished, Fearful, Finicky, Focused*, Frantic, Frustrated, Full*, Greedy, Hesitant, Impatient, Inappetent, Indifferent, Nervous, Picky, Pleased, Protective, Pushy, Relaxed, Restless, Starving, Stressed, Surprised, Tense, Upset, Vocal, Worried

Supplementary Table S3. Core item set used for the hospital client study and for instrument development grouped by item type.

Day items (n=21)	Mealtime items (n=15)
Active, Affectionate, Athletic, Depressed, Energetic, Fearful, Fun-loving, Happy, Lame, Lazy, Loving, Nervous, Painful, Pleased, Sad, Scared, Sleepy, Stiff, Tired, Wobbly, Worried	Aggressive, Bored, Calm, Cheerful, Curious, Disinterested, Excited, Famished, Fearful, Nervous, Picky, Protective, Starving, Stressed, Tense

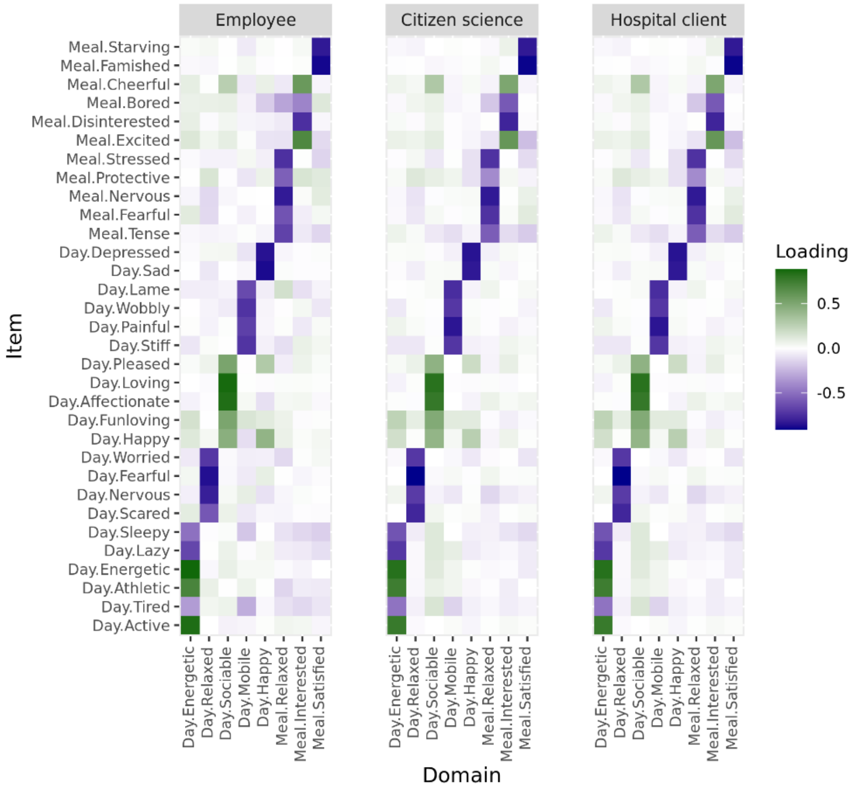

Supplementary Figure S1. Instrument factor loadings and domain mapping obtained for an 8-factor analysis performed on the three studies performed. Item and domain prefixes day and meal refer to daytime and mealtime, respectively.

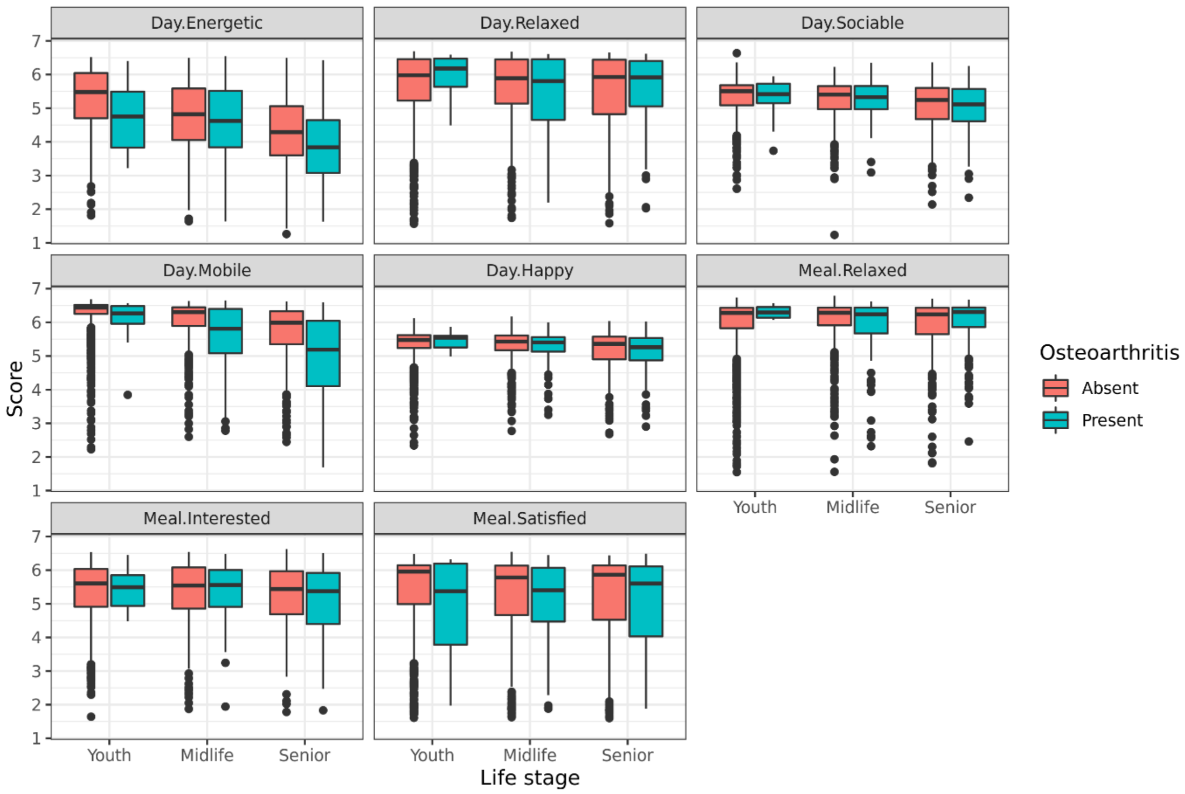

Supplementary Figure S2. Effect of life stage and osteoarthritis disease status on domain scores. Domain prefixes day and meal refer to daytime and mealtime, respectively.
